# Molecular phylogeny of Oncaeidae (Copepoda) using nuclear ribosomal internal transcribed spacer (ITS rDNA)

**DOI:** 10.1371/journal.pone.0175662

**Published:** 2017-04-25

**Authors:** Iole Di Capua, Fulvio Maffucci, Raimondo Pannone, Maria Grazia Mazzocchi, Elio Biffali, Alberto Amato

**Affiliations:** 1 Department of Integrative Marine Ecology, StazioneZoologica Anton Dohrn, Villa Comunale Naples–Italy; 2 Department of Research Infrastructures for Marine Biological Resources, Aquarium Unit, StazioneZoologica Anton Dohrn, Villa Comunale Naples–Italy; 3 Department of Research Infrastructures for Marine Biological Resources, Molecular Biology and Bioinformatics Unit, StazioneZoologica Anton Dohrn, Villa Comunale Naples–Italy; University of Connecticut, UNITED STATES

## Abstract

Copepods belonging to the Oncaeidae family are commonly and abundantly found in marine zooplankton. In the Mediterranean Sea, forty-seven oncaeid species occur, of which eleven in the Gulf of Naples. In this Gulf, several *Oncaea* species were morphologically analysed and described at the end of the XIX century by W. Giesbrecht. In the same area, oncaeids are being investigated over seasonal and inter-annual scales at the long-term coastal station LTER-MC. In the present work, we identified six oncaeid species using the nuclear ribosomal internal transcribed spacers (ITS rDNA) and the mitochondrial cytochrome c oxidase subunit I (mtCOI). Phylogenetic analyses based on these two genomic regions validated the sisterhood of the genera *Triconia* and the *Oncaea sensu stricto*. ITS1 and ITS2 phylogenies produced incongruent results about the position of *Oncaea curta*, calling for further investigations on this species. We also characterised the ITS2 region by secondary structure predictions and found that all the sequences analysed presented the distinct eukaryotic hallmarks. A Compensatory Base Change search corroborated the close relationship between *O*. *venusta* and *O*. *curta* and between *O*. *media* and *O*. *venusta* already identified by ITS phylogenies. The present results, which stem from the integration of molecular and morphological taxonomy, represent an encouraging step towards an improved knowledge of copepod biodiversity: The two complementary approaches, when applied to long-term copepod monitoring, will also help to better understanding their genetic variations and ecological niches of co-occurring species.

## Introduction

Copepods are the most abundant metazoans in marine zooplankton represented by thousands of species. In the late XIX century, Giesbrecht established the family Oncaeidae in a monography on pelagic copepods from the Gulf of Naples (Western Mediterranean Sea, GoN) and described numerous *Oncaea* species [1]. The taxonomic position of Oncaeidae is still under debate as some authors allocate this family within the order Cyclopoida [2,3] while others within Poecilostomatoida [4]. The genus level classification is also debatable. According to Boxshall and Halsey [2], Oncaeidae family contains seven genera (*Archioncaea*, *Conaea*, *Epicalymma*, *Monothula*, *Oncaea*, *Spinoncaea*, *Triconia*); however, Heron and Frost [5] rejected the genus *Triconia*. In the last seven decades, several oncaeids were described from the Mediterranean Sea [6–10]. Among the total 47 species recorded in the basin (including 33 *Oncaea* and 9 *Triconia* species) [3,11], only six are considered to be endemic [3]. In the GoN, three genera occur regularly: *Oncaea* with five species (*O*. *curta*, *O*. *media*, *O*. *mediterranea*, *O*. *scottodicarloi*, *O*. *venusta*), *Triconia* with five species (*T*. *conifera*, *T*. *dentipes*, *T*. *hawii*, *T*. *minuta*, *T*. *rufa*) and *Monothula subtilis* (M. G. Mazzocchi and I. Di Capua, unpublished data).

Oncaeidae are characterised by having a cyclopiform body, five-segmented prosome and urosome, dorsally located genital apertures and caudal rami with six setae [2]. These diagnostic characters, although well established and widely used for taxonomic identification, are often insufficient to distinguish species that appear very similar. Giesbrecht’s species descriptions were accompanied with highly detailed Indian-ink drawings of morphological characters along with a useful dichotomous key [1] that is still of great utility for taxonomists. In recent years, further morphological studies have been carried out to improve oncaeid taxonomy [12,13]. Difficulties in oncaeid identification require advanced taxonomic expertise. Integrated taxonomy seems to be a very useful tool for implementing oncaeid systematics but is still at its early stage [14]. A correct morphological identification of specimens is crucial for a sound integrated approach before molecular characterisation and phylogenetic reconstructions are addressed. Indeed, taxonomic studies have shown that many apparently well-known species, such as *T*. *conifera* and *O*. *media*, are complexes of closely related, yet distinct, species [5,10,13,15]. Molecular taxonomy can also improve and refine our knowledge of the ecology of this copepod family, which is at the moment limited to a few studies [16–18].

The aim of the present work is to identify, using a molecular approach, oncaeid species that occur in the type locality where they were morphologically described more than a century ago [1]. The usefulness of ribosomal regions of the nuclear DNA (rDNA) to infer phylogenetic positions in cyclopoid [19,20] and calanoid copepods [21–25] has been recently demonstrated. Nevertheless, comparison of phylogenetic trees based on different markers can be used to identify evolutionary processes, e.g., possible events of reticulate evolution [20,23,26]. For this reason, we analysed also the mitochondrial cytochrome c oxidase subnit I sequence (COI mtDNA). This marker is widely used for differentiating populations and identifying cryptic species within copepods [22,23,27–32] and for barcoding [33–40]. Moreover, the use of mtCOI allowed us to validate our analyses and compare them with previous findings. We extended our investigation to include internal transcribed spacers of the nuclear ribosomal cistron (ITS rDNA) to define the taxonomic status and genetic relatedness of species within the family Oncaeidae. To the best of our knowledge, the present work is the first phylogenetic study carried out with a nuclear ribosomal marker on multiple oncaeid species (but see ref. [41]). By *in silico* prediction of the ITS2 secondary structure, we characterised this region strengthening our phylogenetic analyses. Noteworthy, we extracted genomic DNA from single individuals making available mitochondrial and nuclear sequences for the same specimen. With the present study, we aimed at implementing ITS rDNA phylogeny in copepod studies from the Mediterranean Sea, a biodiversity hotspot for marine fauna. The previous morphological [42–44] and molecular [19] research on the freshwater cyclopoid genus *Mesocyclops* inspired our thorough investigation of oncaeid copepods in the Mediterranean Sea.

## Materials and methods

### Sampling and morphological identification

Zooplankton samples were collected in winter 2016 at station LTER-MC [45] in the GoN. Vertical hauls were performed from -50 m to the surface with a WP2 net (0.25 m^2^ mouth area, 200 μm mesh size). The sampling period, from January to March 2016, corresponds to the period of all oncaeid species co-occurrence in the GoN. Copepods are neither endangered nor protected species, they are not included in the list of human food resources, hence no specific permissions are required to collect copepods in Italy. The LTER-MC is a long term monitoring station located two miles offshore (40°48.5’N, 14°15’E) close to the 80 m isobath [45]. The Stazione Zoologica Anton Dohrn carries out regular sampling at this station since January 1984 [45]. No permissions are needed to sample at the LTER-MC station for employees of the Stazione Zoologica. Physico-chemical and biological data characterising the pelagic system at LTER-MC are reported in ref. [45]. Data collected at LTER-MC are weekly updated on the website http://szn.macisteweb.com.

In the laboratory, adult female oncaeids were individually sorted from the live sample under a stereo-microscope Leica M 165 C (Leica Microsystems Srl, Milan, Italy) and identified at the species level. The characters used for species identification are summarised in the dichotomic key reported by Di Capua and Boxshall [11] and in Table 1. The total body length of all specimens used in the present study was measured under a stero-microscope from the tip of prosome to the distal end of the caudal rami in dorsal position; further morphometric analyses of total body and diagnostic characters were examined at the scanning electron microscope (SEM) following Di Capua and Boxshall [11] (Table 1).

**Table 1:** Morphological diagnostic characters used to discriminate the 11 species of Oncaeidae present in the Gulf of Naples (*measured using traditional method). The species investigated in the present work are indicated in bold. List of characters [11]: 1 = body size **≥** 1mm; 2 = body size **<** 1mm; 3 = exoskeleton **moderately** chitinised; 4 = exoskeleton **heavily** chitinised; 5 = leg 4 endopod **with** distal conical process; 6 = leg 4 endopod **without** distal conical process; 7 = prosome **with** conspicuous dorso-posterior projection in lateral view; 8 = prosome **without** conspicuous dorso-posterior projection in lateral view; 9 = prosome to urosome ratio; 10 = genital double-somite length to width ratio; 11 = anal somite length to width ratio; 12 = caudal ramus length to width ratio.

Genus	species (n) (* (*Total Length ± σ [mm])	1	2	3	4	5	6	7	8	9	10	11	12
*Oncaea*	***venusta*** (8) (1 ± 0.1)	**☑**			**☑**		**☑**		**☑**	**1.8**	**1.3**	**1.8**	**3.0**
	***mediterranea*** (5) (0.8 ± 0.1)		**☑**		**☑**		**☑**		**☑**	**1.9**	**1.9**	**0.8**	**3.0**
	***scottodicarloi*** (5) (0.6 ± 0.1)		**☑**	**☑**			**☑**		**☑**	**2.8**	**1.6**	**0.8**	**3.0**
	***media*** (3) (0.7 ± 0.1)		**☑**	**☑**			**☑**		**☑**	**2.6**	**1.6**	**0.7**	**2.6**
	***curta*** (3) (0.5± 0.1)		**☑**		**☑**		**☑**		**☑**	**2.5**	**1.5**	**0.6**	**3.0**
*Triconia*	*conifera*	**☑**			**☑**	**☑**		**☑**		2.8	1.6	1.5	1.0
	*dentipes*		**☑**	**☑**		**☑**			**☑**	1.9	1.6	1.0	1.6
	*minuta*		**☑**	**☑**		**☑**			**☑**	2.3	1.6	0.7	1.7
	*umerus*		**☑**		**☑**	**☑**			**☑**	2.4	1.5	0.5	1.3
	***hawii*** (1) (0.7)		**☑**	**☑**	**☑**	**☑**				**2.7**	**1.6**	**0.8**	**1.7**
*Monothula*	*subtilis*		☑	☑			☑		☑	2.0	1.3	0	0

### DNA extraction, amplification and sequencing

Total genomic DNA was extracted from single fresh individuals of pre-identified morphospecies with NucleoSpin® Tissue kit (Machery-Nagel GmbH & Co. KG, Düren, Germany) following the manufacturer’s instructions. PCR fragments were amplified from the mitochondrial cytochrome c oxidase subunit I (COI mtDNA) and the nuclear ribosomal complex including the internal transcribed spacers 1 and 2 (ITS1-5.8S-ITS2 rDNA) using primer pairs LCO1490/HCO2198 [46] and ITS1/Sp1-5 [47,48], respectively.

All PCR reactions were carried out in 25 μl volumes containing 1× PCR reaction buffer (Roche Molecular Systems, Inc), 0.2 mM of each dNTP, 1 μM of each primer, 2.5 U of Taq DNA Polymerase (Roche Molecular Systems, Inc) and approximately 5 to 10 ng of genomic DNA. PCR cycling parameters were as follows: initial denaturation at 94°C for 300s, followed by 40 cycles of 94°C for 60 s, the respective annealing temperature (45°C for COI, 58°C for ITS) for 60 s and 72°C for 60 s, followed by a final extension of 72°C for 420s. Negative controls were included in each batch of PCR amplifications to detect contamination. 5 μl of the PCR products (710 bp long for COI and 950 bp long for ITS) were checked by agarose gel electrophoresis and produced single bands at the expected size. Amplicons were purified using High Pure PCR Product Purification Kit (Roche Diagnostic GmbH, Mannheim, Germany).

Purified PCR products were cloned with Invitrogen® TOPO® TA Cloning® kit (ThermoFisher Scientific, Waltham, MA, USA) and transformed into One Shot® TOP10 Competent Cells (ThermoFisher Scientific, Waltham, MA, USA) following the manufacturer’s instructions. Positive transformants carrying the insert of the expected size were identified by PCR screening using the primer pair T7/M13rev. Plasmid DNA from positive colonies was isolated using GenElute® Plasmid Miniprep Kit (Sigma-Aldrich S.r.l. Milan, Italy) and both strands of the insert were sequenced with primers T7 and M13rev (3 individual clones per PCR product).

Sequence reactions were obtained with the BigDye Terminator Cycle Sequencing technology (Applied Biosystems, Foster City, CA, USA), purified in automation using the AgencourtCleanSEQ Dye terminator removal Kit (Agencourt Bioscience Corporation, Beverly, MA, USA) and the robotic station Freedom Evo 200 (TecanTecan Group Ltd. Switzerland). Products were analysed by Capillary Electrophoresis using the 3730 DNA Analyzer (Applied Biosystems, ThermoFisher Scientific, Waltham, MA, USA).

Forward and reverse chromatograms of each sample were visualised and assembled using the software package SeqManII (DNASTAR Inc., Madison, WI, USA).

### Phylogenetic analyses

Sequences obtained from our specimens and from GenBank (Table 2) were imported in Bioedit Sequence Alignment Editor 7.0.9.0 [49] software and first automatically aligned by ClustalW then manually refined. Maximum Likelihood and Bayesian inference were carried out on three sequence alignments; ITS1-5.8S-ITS2, ITS1, and COI. For all the alignments Modeltest [50] implemented in the software MEGA7.0.18 [51] was run in order to find the best evolutionary model that fitted the dataset. For ITS phylogenies Tamura-Nei [52] model was applied. A discrete Gamma distribution was used to model evolutionary rate differences among sites. For ITS1-5.8S-ITS2, 19 DNA sequences for a total of 640 positions, including gaps, were considered for the analyses. For ITS1, 23 sequences and 329 positions were analysed. The COI analysis was performed using General Time Reversible [53] model with Gamma distribution and invariable sites (GTR+G+I).The analysis involved 100 nucleotide sequences, of which nine from outgroup insect species. The outgroups were chosen by blasting the more divergent ingroup COI sequence in GenBank and retreiving the non-copepod best hits. Codon positions included were 1st+2nd+3rd+Noncoding. All positions containing gaps and missing data were eliminated, for a total of 483 positions in the final dataset. For all the analyses, initial trees for the heuristic search were obtained by applying the Neighbor-Joining method to a matrix of pairwise distances estimated using the Maximum Composite Likelihood (MCL) approach. 10,000 bootstrap replications were performed for each phylogenetic analysis. The resulting trees were analysed and edited in MEGA7.

**Table 2:** List of all the sequences used for phylogenetic analyses. Sequences produced in this study are reported in bold. Species name reported in GenBank entry, voucher number or isolate name and GenBank accession number are reported for each COI, ITS and ITS1 entries. The last nine rows contain information for the COI outgroup sequences.

species	Voucher or isolate	COI	Voucher or isolate	ITS^a^	Voucher or isolate	ITS1 only^b^
*Copilia mediterranea*	CMD1	KT429931^c^				
*Copilia mirabilis*	Cop_sp47-1	EU856805^c^				
	comi3	HM045305^c^				
	cami	HM045363^c^				
	qj10	HM045375^c^				
	qj2	HM045376^c^				
	qj9	HM045408^c^				
*Corycaeus affinis*	coaf1	HQ718595^c^				
	coaf2	HQ718596^c^				
	coaf3	HQ718597^c^				
*Cyclops insignis*			-	KF153690		
*Cyclops kolensis*			-	KF153689		
*Cyclops strenuus*			-	KF153691		
*Diacyclops bicuspidatus*			-	KF153697		
*Ditrichocorycaeus anglicus*	MT00597	KT208395^d^				
	MT03918	KT208535^d^				
	MT00599	KT208842^d^				
	MT03917	KT208955^d^				
	MT03913	KT209148^d^				
	MT00771	KT209282^d^				
	MT00598	KT209415^d^				
	MT03914	KT209522^d^				
	MT00596	KT209568^d^				
*Farranula gibbula*	FG1	KM114216^c^				
		KP985538^c^				
*Macrocyclops albidus*			-	KF153696		
*Macrocyclops distinctus*			-	KF153695		
*Megacyclops viridis*			isolate_1	KF153698		
			isolate_2	KF153699		
*Mesocyclops leuckarti*			-	KF153692		
*Oithona similis*	p36ois	EU599542^c^		KF153700		
	p36ois	EU599543^c^				
	p36ois	EU599544^c^				
	HY_Os003	JN230859^c^				
	HY_Os004	JN230860^c^				
	HY_Os005	JN230861^c^				
	HY_Os006	JN230862^c^				
	HY_Os007	JN230863^c^				
	HY_Os008	JN230864^c^				
	HY_Os010	JN230865^c^				
	HY_Os011	JN230866^c^				
	HY_Os012	JN230867^c^				
	HY_Os013	JN230868^c^				
	HY_Os014	JN230869^c^				
	HY_Os015	JN230870^c^				
	HY_Os001	JN230885^c^				
	HY_Os002	JN230886^c^				
	MT00715	KT208459^c^				
	MT00710	KT208745^c^				
*Oncaea curta*	**Oc1GoN**	**KX650376**	**Oc1GoN**	**KX620518**		
*Oncaea media*	OM2	KT369530^c^	**Omi1GoN**	**KX620519**	C26	AM114421
*Oncaea mediterranea*	rjm1258	AB457134^e^	**Om1GoN**	**KX620520**		
*Oncaea* cf. *mediterranea*	rjm1254	AB457130^e^				
*Oncaea parabathyalis*	rjm1269	AB457147^e^				
*Oncaea prendeli*	rjm1267	AB457146^e^				
*Oncaea scottodicarloi*	rjm1256	AB457132^e^				
	rjm1257	AB457133^e^				
	**Osdc1GoN**	**KX650375**	**Osdc1GoN**	**KX620521**		
*Oncaea shmelevi*	rjm1265	AB457145^e^				
*Oncaea venusta*			**Ov1GoN**	**KX620522**	C8	AM114420
					C15	AM114418
					C9	AM114419
*Oncaea waldemari*	rjm1259	AB457136^e^				
*Oncaea* sp.			MVZ-2013	KF153701		
*Oncaea* sp. 7	rjm1260	AB457138^e^				
*Oncaea* sp. 7	rjm1261	AB457139^e^				
*Pachos punctatum*	papu	HM045399^c^				
*Sapphirina angusta*	Co041.1.1	GU171328^f^				
	SANG1	KT345967^c^				
	SANG2	KT345968^c^				
*Sapphirina bicuspidata*	SBC1	KT354291^c^				
	SBC2	KT354292^c^				
*Sapphirina darwinii*	sada1	HM045389^c^				
*Sapphirina metallina*	same	HM045344^c^				
	M3090	KF985240^h^				
	SM1	KT429933^c^				
	SM3	KU144690^c^				
	SM4	KU144691^c^				
	SM5	KU200948^c^				
*Sapphirina opalina*	saop	HM045409^c^				
	saop2	HM045410^c^				
	SAOP3	HM045411^c^				
	saop4	HM045412^c^				
	SO1	KU158879^c^				
	SO2	KU158880^c^				
	SO3	KU158881^c^				
	SO4	KU158882^c^				
	SO5	KU158883^c^				
*Sapphirina scarlata*	sasc	HM045348^c^				
	SSR1	KT351342^c^				
	SSR2	KT351343^c^				
	SSR3	KT351344^c^				
*Sapphirina stellata*	SSTL1	KT354294^c^				
*Stellicola* sp.		DQ889130^g^				
*Thermocyclops crassus*			-	KF153694		
*Thermocyclops oithonoides*			-	KF153693		
*Triconia conifera*	rjm1271	AB457148^e^				
	rjm1272	AB457149^e^				
*Triconia dentipes*	rjm1260	AB457137^e^				
*Triconia elongata*	rjm1253	AB457129^e^				
*Triconia hawii*			**Th1GoN**	**KX620523**		
*Triconia minuta*	rjm1265	AB457142^e^				
	rjm1264	AB457143^e^				
	rjm1265	AB457144^e^				
*Triconia umerus*	rjm1262	AB457140^e^				
	rjm1263	AB457141^e^				
*Anopheles pristinus*	SP53_101	GU989357				
	SP55_2	GU989358				
	VP11a	GU989348				
*Gressittacantha terranova*	TNT1e_a	HM461319				
	TNT2c_i	HM461301				
	TNT2c_b	HM461312				
	TNT2c_a	HM461287				
*Lepicerus inaequalis*		KJ871320				
*Mycetaulus bipunctatus*	BIOUG03450-D01	KR436825				

^a^ref. [20].

^b^ref. [41].

^c^ direct submission.

^d^ref. [40].

^e^ref. [10].

^f^ref. [36].

^g^ref. [55].

^h^ref. [56].

Using the same alignments, Bayesian inferences were carried out. The analyses were forced to jump among the evolutionary models for nucleotide sequence alignment implemented in MrBayes 3.2 [54]. The following settings were applied to Bayesian phylogenetic analyses for all the alignments. Two parallel and completely independent Markov Chain Monte Carlo (MCMC) runs were carried out on data matrices. Three hot and one cold chain drove the analyses. The number of generations was set to 10 million and the sampling frequency at 100 generations. The first 25% of the samples from the cold chain were discarded. Consensus trees, with posterior probability of each node and branch lengths, are reported here after a 50% majority-rule consensus phylogeny. Phylogenetic trees were visualised and edited in the FigTree (Tree Figure Drawing Tool Version 1.4.2) software (http://tree.bio.ed.ac.uk/).

### ITS2 secondary structure reconstructions

The last 400 bp of all the ITS sequences were scanned for secondary structure prediction for two reasons: 1) to identify the exact margins of this region, as in GenBank the entry descriptions for poecilostomatoid ribosomal sequences fail to define regions structurally; 2) because the ITS2 secondary structures can be used for phylogenetic purposes together with the sequence itself. RNA secondary structure predictions were performed using mfold software [57] and visualised in PseudoViewer 3 Web Application (http://pseudoviewer.inha.ac.kr/).

#### ITS2 alignment and phylogeny

Based on secondary structure reconstructions, the ITS2 region of all the sequences available were manually aligned in BioEdit and then ML (Hasegawa-Kishino-Yano (HKY [58]) +G model, 10,000 bootstrap replications) and Bayesian inference were carried out. For Bayesian inference, the ‘doublet model’ implemented in MrBayes was used in order to merge sequence information and secondary structure in the same analysis. This analysis considers the stem regions and weighed compensatory base changes (CBCs) and hemi-CBCs (HCBCs) differently. Eight chains and 30 million generations were set to run the analysis.

## Results

### Oncaeids in the Gulf of Naples

Among the eleven Oncaeidae species occurring regularly in the Gulf of Naples, we selected for this study five *Oncaea* species (*Oncaea curta*, *O*. *media*, *O*. *mediterranea*, *O*. *scottodicarloi*, *O*. *venusta*) and one *Triconia* species (*T*. *hawii*) that are the most abundant and widely distributed in Mediterrenean Sea from coastal to open waters. Results of the morphometric analyses and the diagnostic features are summarised in Table 1.

#### COI phylogeny

**Fig 1:**
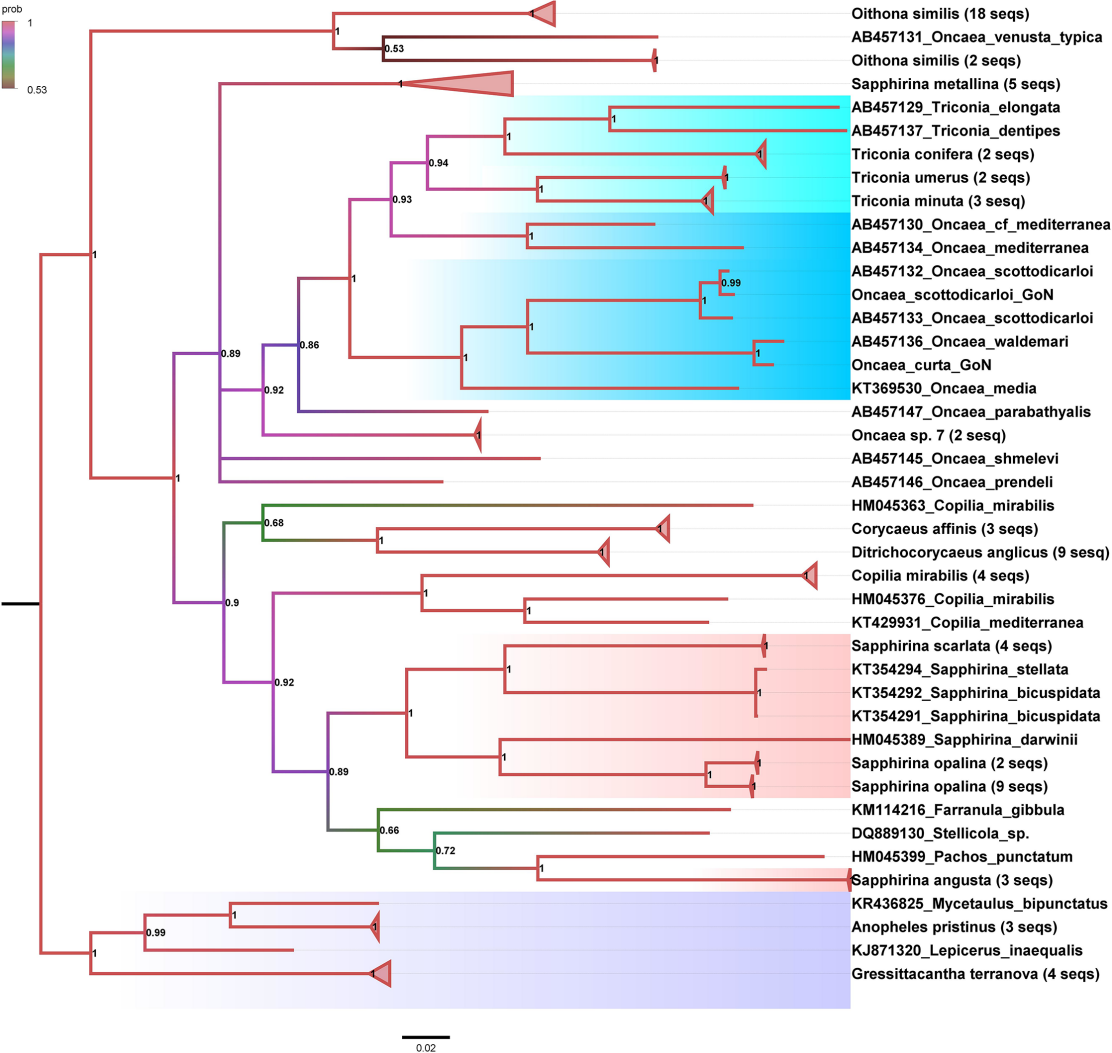
COI phylogeny (split frequency σ = 0.002). Posterior Probability (PP) is reported at each node. Branch colour represents PP; colour code for PP is reported in the figure. Scale bar represents 0.02 substitutions per site. GenBank accession numbers are reported, followed by the genus and species names. If a branch was condensed, the genus and species names are reported followed by the number of sequences contained in each branch. The uncondensed Bayesian tree is reported in S3 Fig. Torquoise and cyan shades indicate the *Triconia*/*Oncaea s*. *s*. clade. The pink shade indicates the *Sapphirina* clades. The lilac shade indicates outgroups. GoN = Gulf of Naples, i.e. sequences produced in the present work.

A 710 bp fragment of the COI was obtained from individuals belonging to *Oncaea curta* and *O*. *scottodicarloi* (Table 2). The COI phylogenies produced in Maximum Likelihood (ML) and by Bayesian inference (BI) presented a prominent topological similarity. The BI resolved more robustly all the clades both at the species and supraspecies levels (Fig 1).

All epipelagic species of *Oncaea s*. *s*. [13] clustered together with *Triconia* clades, shaded in cyan and turquoise (Fig 1and S1 Fig), in a moderately supported clade (posterior probability, PP 0.86 ML 50). *O*. *scottocarloi* from the GoN clustered together with other conspecifics from the Western Mediterranean Sea. *O*. *curta* from the GoN presented 98% sequence identity with *O*. *waldemari* and indeed the two species robustly clustered together (PP 1.00 ML 100). The *Oncaea* cf. *mediterranea* (AB457130) and *O*. *mediterranea* (AB457134) sequences robustly clustered together with *Triconia* (PP 0.93 ML 99). The mesopelagic species *Oncaea* sp. 7 and *O*. *parabathyalis* clustered in basal position to the *Triconia*/*Oncaea s*. *s*. clade for BI; in ML (S1 Fig), the three sequences (AB457138, AB457139, AB457147) produced a weakly supported (ML 56), unstructured clade. Other mesopelagic species, i.e., *O*. *shmelevi* and *O*. *prendeli*, produced unstructured branches in both BI and ML. Highly suprising was the position of *O*. *venusta*: in our BI, the sequence AB457131 clustered with *Oithona similis* with extremely low support (PP 0.53). In ML (S1 Fig), this sequence robustly clustered (ML 100) in a basal position to the *O*. *similis* clade.

*Sapphirina* genus resulted polyphyletic in our analyses with five species clustering in one robust clade (PP 1.00; pink shaded clade), but eight more sequences from specimens identified as *S*. *metallina* and *S*. *angusta* clustered in separate clades. In ML, *S*. *bicuspidata*, *S*. *stellata*, *S*. *darwini* and *S*. *opalina* clustered together (ML 62), while *S*. *scarlata*, *S*. *angusta* and *S*. *metallina* clustered in unresolved separate clades. Another incongruence was the sequence from *Copilia mirabilis* (HM045363) that weakly clustered (PP 0.68) with coryceid species *Corycaeus affinis* and *Ditricorychaeus anglicus* in BI. In ML, this sequence produced an unstructured branch. This finding can either be due to a mis-identification of the *Copilia* specimen or to a peculiar COI sequence present in this particular isolate. In ML, *Copilia mirabilis*/*mediterranea*, *Ditricorycaeus* and *Corycaeus* clades were not resolved, in BI they clustered in basal position to the *Sapphirina*/*Farranula*/*Stellicola*/*Pachos* clade.

#### ITS phylogenies

**Fig 2:**
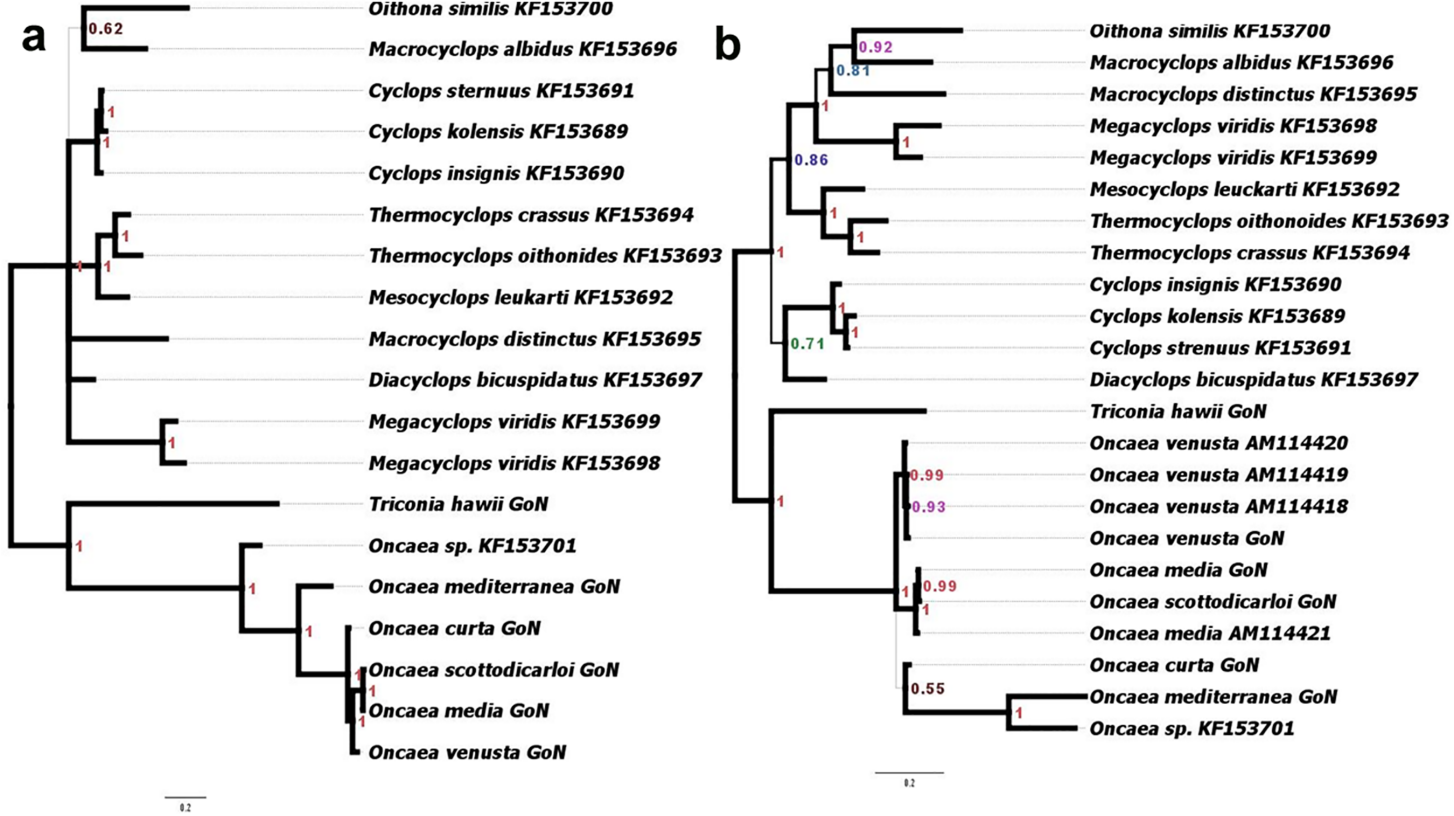
a. ITS1-5.8S-ITS2 (split frequency σ = 0.003) and b. ITS1 (split frequency σ = 0.003) phylogenetic trees reconstructed by Bayesian inference. Posterior Probability (PP) is reported at each node. Thickness of branches represents PP. Scale bar represents 0.2 substitutions per site. The species name and the GenBank identification number of the corresponding sequence are reported. GoN = Gulf of Naples, i.e. sequences produced in the present work.

A 950 bp fragment of the ITS1-5.8S-ITS2 region was obtained from six species collected at LTER-MC station: *Oncaea curta*, *O*. *media*, *O*. *mediterranea*, *O*. *scottodicarloi*, *O*. *venusta*, and *Triconia hawii* (Table 2). ITS phylogeny carried out by BI (Fig 2A) and ML (S2 Fig) presented the same tree topology, with Bayesian analysis much more supported than ML. For this reason only Bayesian tree is presented (Fig 2A). In these analyses, our *Triconia* sequence (the first full length ITS sequence for the genus) clustered as sister taxon to *Oncaea s*. *s*. [13]. For these analyses, only cyclopoid sequences were used as outgroup because no poecilostomatoid ITS sequences were available. All cyclopoid species clustered together in a separate clade. Bayesian posterior probability of all but one node was 1.00 revealing the robustness of the analyses, much higher than ML (S2 Fig). ITS1-based phylogeny was carried out to include as many *Oncaea* sequences as possible (for some species in GenBank only ITS1 sequences are deposited) for a wider comparison (Fig 2B). For this genomic region, BI (Fig 2B) and ML (S3 Fig) did not show topological congruence for the position of *Triconia* that in one case showed sisterhood with *Oncaea* spp. (PP 1.00, Fig 2B), while in ML this taxon clustered in a basal position to the other cyclopoids (bootstrap support 45, S3 Fig). The Bayesian tree topology recalled that produced using ITS1-5.8S-ITS2 region, with *Triconia* as a sister clade to *Oncaea s*. *s*. [13]. The different *O*. *venusta* sequences clustered together with *O*. *venusta* from the GoN and presented very low level of variability. Mean sequence identity among *O*. *venusta* from the GoN and the other representatives of this species was 98.7%, while identity among the three other ITS1 sequences was 99.5%.

#### ITS2 secondary structures

ITS2 was also analysed for RNA secondary structure prediction. The first significant result obtained by secondary structure prediction is that ITS2 varies in length in the different species spanning from 166 bp in *Thermocyclops crassus* to 237 bp in *Macrocyclops distinctus* (Table 3, S4 Fig). Among oncaeids, the longest ITS2 sequence recorded was that of *O*. *mediterranea* (210 bp) while the shortest one was that of *T*. *hawii* (169 bp) (Table 3, S4 Fig). All the sequences analysed presented the distinct eukaryotic hallmarks: i. four main helices, ii. helix III as the longest one; iii. the presence of a characteristic motif at the apex of helix III, and iv. a pyrimidine-pyrimidine mismatch in helix II [59].

**Table 3:** Length in base pair of the ITS2 regions inferred from secondary structure predictions. Species reported in bold were produced in the present study.

Species	ITS2 lenght
***Oncaea curta***	172
***Oncaea media***	183
***Oncaea mediterranea***	210
***Oncaea scottodicarloi***	183
***Oncaea venusta***	179
*Oncaea* sp.	175
***Triconia hawii***	169
*Cyclops insignis*	183
*Cyclops kolensis*	184
*Cyclops sternuus*	184
*Diacyclops bicuspidatus*	188
*Macrocyclops albidus*	184
*Macrocyclops distinctus*	237
*Megacyclops viridis* Borok1	178
*Megacyclops viridis* Borok2	182
*Mesocyclops leukarti*	187
*Oithona similis*	179
*Thermocyclops crassus*	166
*Thermocyclops oithonides*	172

**Fig 3:**
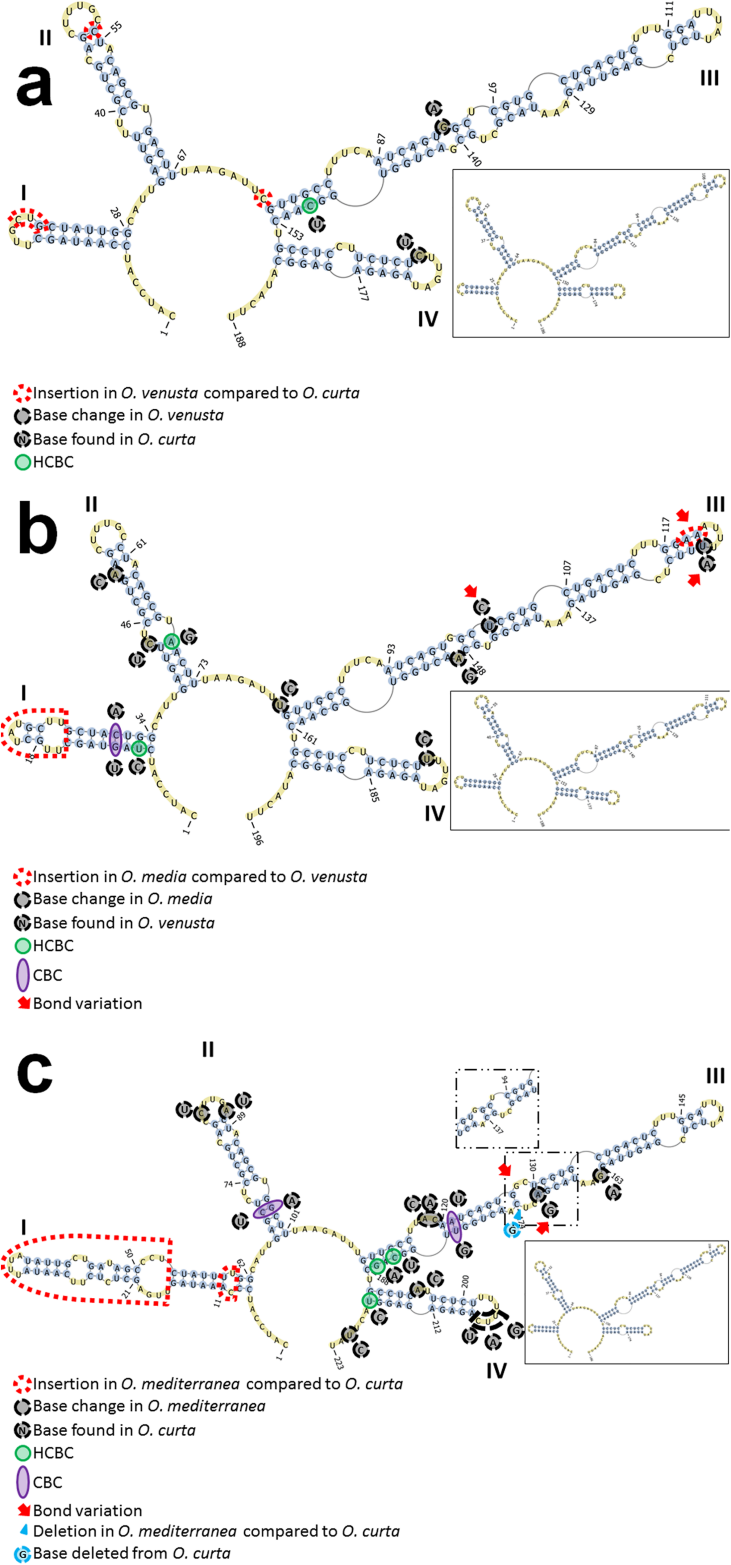
CBC analysis of ITS2 secondary structure for: a. *Oncaea venusta* and *O*. *curta* (*O*. *venusta* structure shown). b. *O*. *media* and *O*. *venusta* (*O*. *media* structure shown). c. *O*. *mediterranea* and *O*. *curta* (*O*. *mediterranea* structure shown).

By CBC search it was possible to corroborate the close relationship between *O*. *venusta* and *O*. *curta* (Fig 3A) and between *O*. *media* and *O*. *venusta* (Fig 3B). Between *O*. *venusta* and *O*. *curta* (Fig 3A) only one HCBC was recorded in helix III with a transition from C to U which produced a non-canonical bond G::U [60]. All the other base changes reported between these two sequences did not produce any structural change to the transcript. This situation makes these two species highly similar with possible vestigial or actual sexual interactions as described in other systems [61]. The comparison between *O*. *media* and *O*. *venusta* (Fig 3B) produced more differences: two HCBCs due to two transitions (U→C in helix I and A→G in helix II), and one CBC involving two transversions in helix I (C→A and G→U). Two insertions were found as well: a huge one at the tip of helix I, which produced a different helix tip; and another one on the 3’ side of the tip of helix III, which created two new A::U bonds. Two non-compensatory base changes were recorded on helix III, a U→C transition and a U→ A transversion. The rest of modifications did not produce more structural changes. The most dramatic changes were found comparing *O*. *mediterranea* to *O*. *curta* (Fig 3C) with two CBCs in helices II and III; three HCBCs (two in helix III and one in helix IV); two insertions in *O*. *mediterranea*, one being very long and producing a much longer helix I compared to *O*. *curta*; two structural changes due to a deletion and an insertion.

#### ITS2 phylogeny based on secondary structure

**Fig 4:**
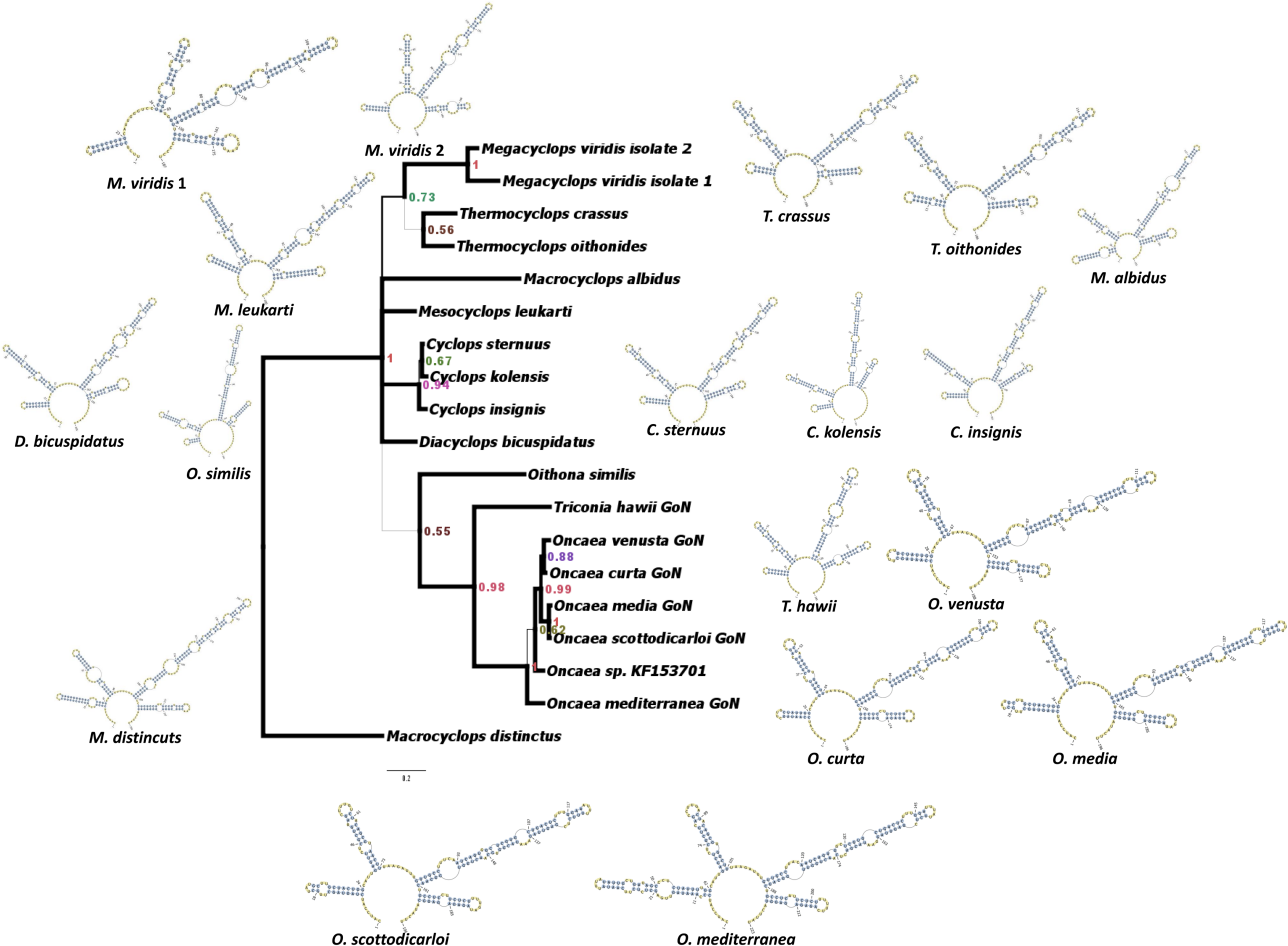
ITS2 stem phylogeny based on Bayesian inference (split frequency σ = 0.002). Posterior Probability (PP) is reported at each node. Thickness of branches represents PP. Scale bar represents 0.2 substitutions per site. ITS2 secondary structure reconstructions are reported for each species. Full-size pictures of secondary structures are reported in S4 Fig.

The phylogenetic tree produced by ML and BI on ITS2 sequence corroborated by secondary structure (see Material and Methods) produced only partially overlapping topologies thus we report the Bayesian tree only (Fig 4). In this analysis, the stem regions were aligned and considered to be homologous. From this tree, *Triconia* and *Oncaea s*. *s*. [13] resulted again as sister taxa with high support (PP = 0.98 ML = 93). Surprisingly, ITS2 region defined a different clustering pattern compared to ITS1 and ITS1-5.8S-ITS2 phylogenies (Fig 2). *O*. *curta* clustered together with *O*. *venusta* with good support (PP = 0.88 ML = 62). This can be explained by the very similar secondary structures displayed by these two species (Fig 3A). The overall topology of ITS2 stem phylogenetic analysis was slightly divergent compared to the rest of the analyses run in the present work, revealing a fairly strong influence of secondary structure in the phylogenetic reconstruction.

## Discussion

Oncaeid copepods are very common and abundant in planktonic communities from neritic areas to open seas and from epipelagic to deep sea. The ecological traits and the role played by oncaeids in the planktonic communities are still poorly known [15–18] and many aspects of their biology are still not completely understood. In the last two decades, numerous studies have increased our knowledge on oncaeid taxonomy and systematics, discussing their potential ecological relevance [12,13,62].

Identification of oncaeids using stereo-microscopy based on morphological features alone is problematic. The advent of molecular techniques has provided researchers with a powerful tool; however the implementation of molecular identification protocols requires information on the genetic diversity of the system of interest [39]. Copepod metabarcoding failed to identify *Oncaea* spp. although microscopic sample counting had revealed their presence [63]. In this paper, we produced sequences from COI, regularly used for species identification [23,27–32] and barcoding [33–40], and ITS rDNA, widely applied for phylogenetic reconstructions in copepods [19–25].

Although the number of available COI sequences for marine planktonic copepods is relatively high, obtaining COI fragments from oncaeids [10,64] using the universal primers [46] commonly employed for copepods proved to be difficult. Additionally, the majority of the COI sequences we retreived from GenBank lack description, discussion and have not been incorporated in phylogenetic reconstructions (Table 2). Oncaeid sequences were not detected from extensive barcoding analysis on Arctic holozooplankton [35], Sargasso Sea zooplankton [36], Northern Sea crustaceans [40], and Southern Korean copepods [65]. It is not clear, however, whether in these works either *Oncaea* or *Triconia* specimens were not collected or sequencing failed to detect them.

In our COI phylogeny, the family Oncaeidae (*Triconia* and *Oncaea*) was monophyletic, but the genus *Oncaea sensu lato* (*s*. *l*.) was not. Polyphyly implies either the existence of one or more homoplasies, or the rise of different species in the same genus by different ancestors. The genus *Triconia* has been rejected by Heron and Frost [5] and our phylogenetic analyses indicate that the validity of this genus is questionable. We suggest the possibility to demote *Triconia* to sub-genus or allocate *Oncaea* sp. 7, *O*. *parabathyalis*, *O*. *shmelevi* and *O*. *prendeli* into another genus. In either case, a re-evaluation of the systematics of this group should be considered. Overall, our COI phylogenetic analyses corroborated the finding of a previous phylogeny carried out on COI and 12S mtDNA sequences obtained from single individuals of *Oncaea* and *Triconia* [10]. In particular, COI identified morphological species quite well, as recently confirmed [65], but the sisterhood of *Triconia* and *Oncaea s*. *s*. was not detected.

The COI sequences of the Mediterranean *O*. *curta*, originally described in Western Ireland [66], have high similarity with *O*. *waldemari* that was described from Southern Brazilian waters [67]. The latter was subsequently identified in the Mediterranean Sea and redescribed with the addition of new morphological characters and molecular support [10]. Nevertheless, before redescription of *O*. *waldemari* [10], no *O*. *curta* sequences were available. Now *O*. *curta* sequences are available (present work), and a detailed morpho-molecular re-evaluation of the species allocation is needed.

*Oncaea venusta* COI sequence produced a very long branch in previous phylogenies (Fig 2 in ref. [10]) indicating high levels of divergence. In our analyses, this sequence clustered together with *Oithona similis*, which is distantly related to *Triconia*/*Oncaea s*. *s*. clade. Moreover, the position of *O*. *venusta* in the 12S phylogeny (Fig 3 in ref. [10]) did not match with COI phylogeny (Fig 2 in ref. [10]). This might be a sign of introgression that produced hybrid speciation. This issue needs more investigation and *ad-hoc* experiments to be properly addressed, but these were beyond the aims of the present study. The paucity of molecular works on oncaeids makes difficult a comprehensive discussion. The present contribution is meant to call attention on this group of copepods, which can be easily overlooked if high-throughput sequencing techniques are applied. Although it was demonstrated that copepod COI region used for barcoding is a good tool for molecular species identification [37], some taxonomic groups may be more challenging than others to be molecularly detected.

ITS is a nuclear multicopy marker part of the ribosomal cistron. This region, although not coding for a protein, is functional to the correct assembly of the mature ribosomes. ITS is transcribed but then it is spliced out the mature ribosome when the large and the small subunits join to the 5.8S. For this reason, this region can accumulate mutations but with certain constraints due to its functional role. ITS is widely used in phylogeny in different systems [19–25,68]. ITS phylogeny of *Oncaea* specimens does not suggest the existence of cryptic species but confirms what previously hypothesised for cyclopoids, i.e. the two internal transcribed spacers in this group of copepods vary at a different pace [20]. The trees produced from ITS1 and ITS2 regions are more resolved than that from ITS1-5.8S-ITS2. The positions of *O*. *curta* in the ITS1 and ITS2 phylogenies are not consistent: in ITS1 it clustered in a clade together with *O*. *mediterranea* and *Oncaea* sp. (MVZ-2013, GenBank KF153701), while in ITS2 phylogeny *O*. *curta* clustered with *O*. *venusta*. We produced the first *O*. *curta* ITS sequence and therefore we could not compare it with previous phylogenies. In all our ribosomal-based phylogenetic analyses, *Triconia hawii* clustered in basal position to the *Oncaea s*. *s*. species, corroborating the sisterhood of these two taxonomic groups. In the case of *Triconia* as well, our *T*. *hawii* ITS sequence is the first produced and future work will elucidate the genetic relationships among species belonging to *Triconia* and *Oncaea*.

Elvers and colleagues [41] identified four morphotypes in *O*. *venusta* based on the body size in different geographic areas [41]. *O*. *venusta* from the GoN clustered in a clade containing both the small and the intermediate morphotypes. Nevertheless, our specimen was 1.0 ± 0.1 mm long (average ± σ) that is the size range reported for the medium-large morph [41]. This finding led us to the conclusion that the sequence variability recorded within the *O*. *venusta* species is not ascribable to sympatric cryptic diversity but to intraspecific variability of the ITS1 region itself. Moreover the sequence identity among the different *O*. *venusta* morphs is extremely high (~99.5%) and this would not justify new species descriptions. The size differences recorded by Elvers and colleagues [41] might be due to morphological plasticity or simply to random intraspecific variations as reported for the cyclopoid *Oithona similis* [69]. The morphological differences (prosome and length of the antennules) of two populations of *O*. *similis* in Barents and White Seas is likely an adaptive response to spatial variation in environmental factors [69].

**Fig 5:**
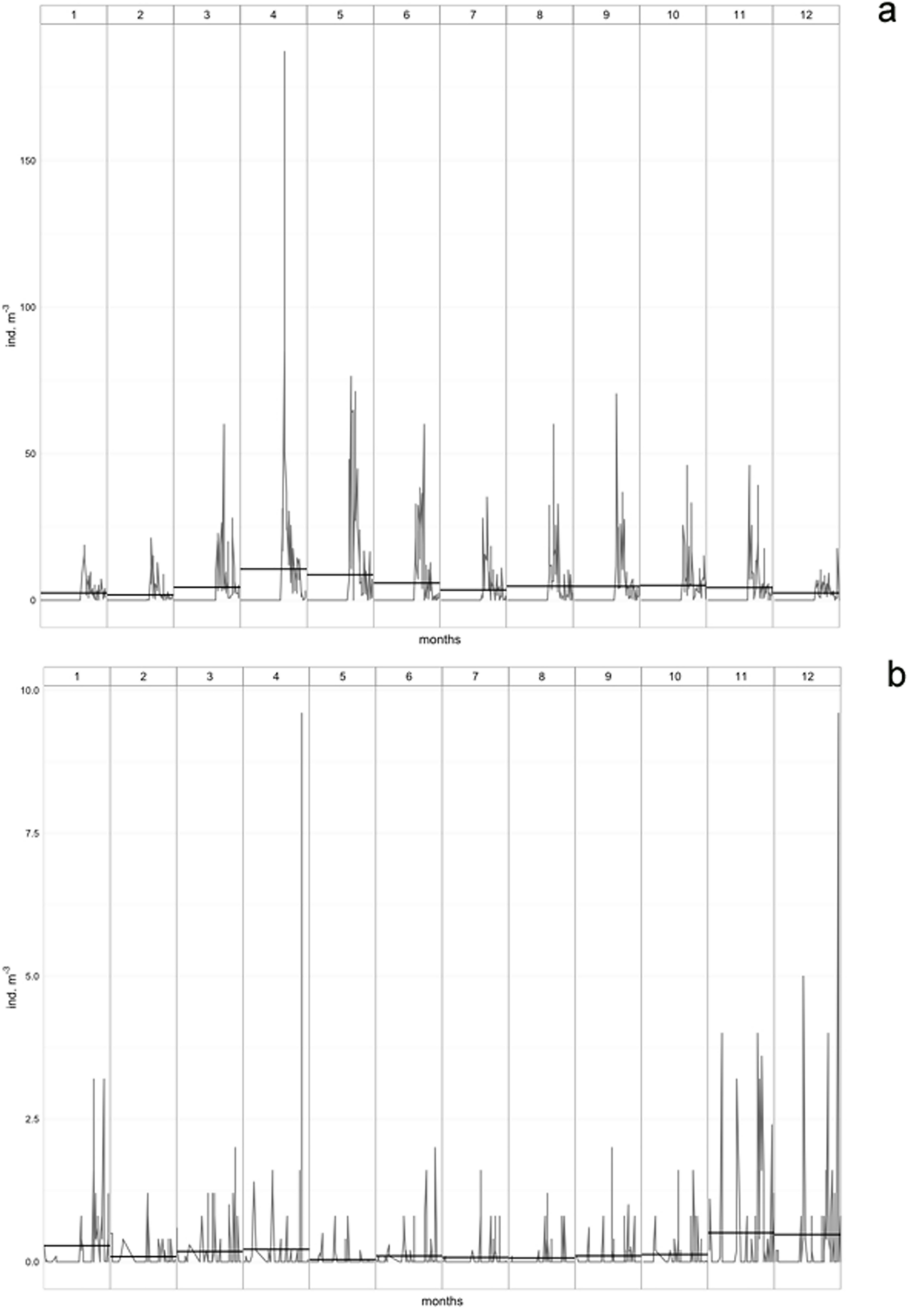
Interannual variability of abundance of a. *Oncaea curta* and b. *Oncaea venusta* recorded at LTER-MC. Thin line shows monthly raw-data (individual abundace) over the period 1984–2015; horizontal bold line represents the interannual monthly-averaged abundaces of the species.

Results of our CBC-search demonstrated that *O*. *venusta* and *O*. *curta* are closely related, showing only one HCBC on the 5’ side at the base of helix III (Fig 3A). In other systems, this ITS2 secondary structure similarity between two entities can be interpreted as a sign of possible vestigial or actual interbreeding ability [61]. Mating compatibility experiments should be carried out in order to verify this hypothesis, as it was done for two populations of the calanoid *Eurytemora affinis* from different localities [27]. The phylogenetic analyses performed on the whole ITS region and on ITS1 only showed *O*. *curta* clustering as sister taxon to the clade grouping *O*. *venusta* and the other species (Fig 2). In the stem phylogenetic analysis carried out on ITS2 only (Fig 4), these two species resulted as sister taxa with a good support. Data obtained at LTER-MC show that *O*. *curta* and *O*. *venusta* differ in abundance and seasonality, with the former peaking mainly in spring and the latter in winter (Fig 5). This may represent an ecological niche differentiation similar to what observed in the calanoid *Clausocalanus* species [70]. The incongruency between ITS1 and ITS2 phylogenies is similar to what previously found in *Mesocyclops leukarti* [20]. Hybrid originof *M*. *leukarti* was advocated [20] and cannot be ruled out for *O*. *curta*, which may have arisen from the hybridisation between common ancestors of *O*. *mediterranea* and *O*. *venusta*. Between *O*. *curta* and *O*. *venusta*, however, the secondary structures are much more similar compared to those predicted for *M*. *leukarti* and *Thermocyclops* or *Macrocyclops*(not shown), possibly revealing a more recent speciation or an incomplete lineage sorting [71]. We used the ITS2 database [72] to reconstruct phylogenetic relationships among *M*. *leukarti*, *Thermocyclops* and *Macrocyclops* (S5 Fig), structurally confirming what reported from the authors [20]. However, more studies are necessary to disentangle this issue.

## Conclusions

The phylogenetic reconstructions carried out with COI mtDNA and ITS1 and ITS2 rDNA corroborate the species identification based on morphological characters. However, the position of *Oncaea curta* was incongruent in the different phylogenies. Moreover, *O*. *curta* and *O*. *waldemari* COI sequences showed high identity. Further detailed work on *O*. *curta* is necessary to clarify the molecular identity and the ecology of this species.

We also characterised the ITS2 region in order to gather as much information as possible from it; ITS2 is widely used in different systems for phylogenetic reconstructions at different levels (genus, species, population, individual) and we propose this region for Oncaeidae phylogenies as in other copepod families [20,73]. Secondary structure predictions and analyses linked to this procedure can shed light on evolutionary history of different species.

Finally, we propose to sequence ITS (or even ITS2 only) from as many copepods as possible in order to broaden our knowledge on these key inhabitants of the pelagic realm and enable future studies based on secondary structure-derived stem phylogeny [59,72,74,75].

## Supporting information

S1 Fig Maximum Likelihood tree constructed on COI mtDNA sequences. The GenBank accession number and the species names are reported. Digits at the nodes indicate bootstrap support (10,000 replicates). Values below 50 are not reported.
(TIF)

S2 Fig Maximum Likelihood tree constructed on ITS (ITS1-5.8S-ITS2) rDNA sequences. Digits at the nodes indicate bootstrap support (10,000 replicates). Values below 50 are not reported.
(TIF)

S3 Fig Maximum Likelihood tree constructed on ITS1 rDNA sequences. Digits at the nodes indicate bootstrap support (10,000 replicates). Values below 50 are not reported.
(TIF)

S4 Fig ITS2 rDNA secondary structure predictions for all the sequences investigated in this work. (DOCX)

S5 Fig Maximum Likelihood tree constructed on ITS2 rDNA sequences from the species *Macrocyclops leukarti*, *Megacyclops albidus*, *Thermocyclops oithonides*, *Oithona similis* and five *Cyclops sternuus* sequences used as outgroup. This tree was built using the ITS2 database [72] facilities.
(TIF)
