# Supplementary figures and images for: Molecular phylogeny of Oncaeidae (Copepoda) using nuclear ribosomal internal transcribed spacer (ITS rDNA)
Source: PLoS One. 2017 Apr 25;12(4):e0175662. doi: 10.1371/journal.pone.0175662 (PMC5404819; doi:10.1371/journal.pone.0175662)

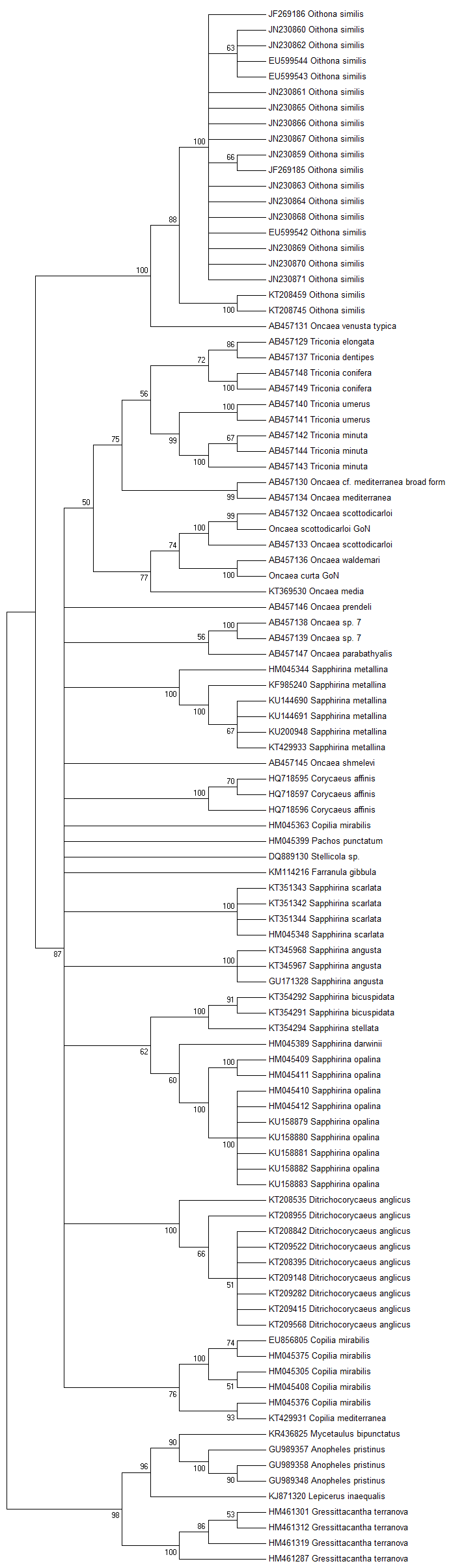

Supplement: S1 Fig — The GenBank accession number and the species names are reported. Digits at the nodes indicate bootstrap support (10,000 replicates). Values below 50 are not reported. (TIF) [file pone.0175662.s001.tif]

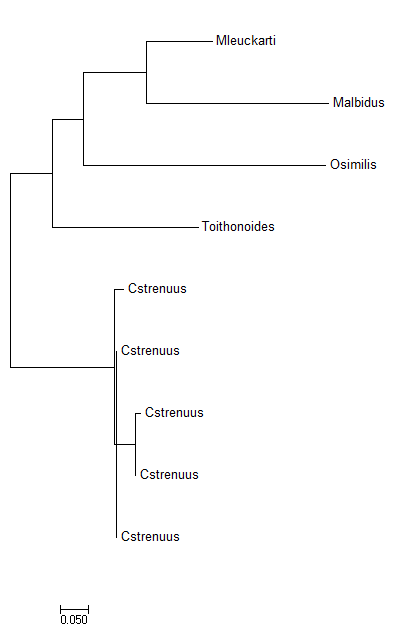

Supplement: S5 Fig — This tree was built using the ITS2 database [72] facilities. (TIF) [file pone.0175662.s005.tif]
