# Supplementary material for: Molecular phylogeny of Oncaeidae (Copepoda) using nuclear ribosomal internal transcribed spacer (ITS rDNA)
Source: PLoS One. 2017 Apr 25;12(4):e0175662. doi: 10.1371/journal.pone.0175662 (PMC5404819; doi:10.1371/journal.pone.0175662)

Supplementary material to the paper:

Molecular phylogeny of Oncaeidae (Copepoda) using nuclear ribosomal Internal Transcribed Spacer (ITS rDNA)

Iole Di Capua, Fulvio Maffucci, Raimondo Pannone, Maria Grazia Mazzocchi, Elio Biffali, Alberto Amato

ITS2 secondary structure of *Oncaea curta*



 ITS2 secondary structure of *Oncaea media*





ITS2 secondary structure of *Oncaea mediterranea*





ITS2 secondary structure of *Oncaea scottodicarloi*





ITS2 secondary structure of *Oncaea venusta*





ITS2 secondary structure of *Triconia hawii*





ITS2 secondary structure of *Cyclops insignis*


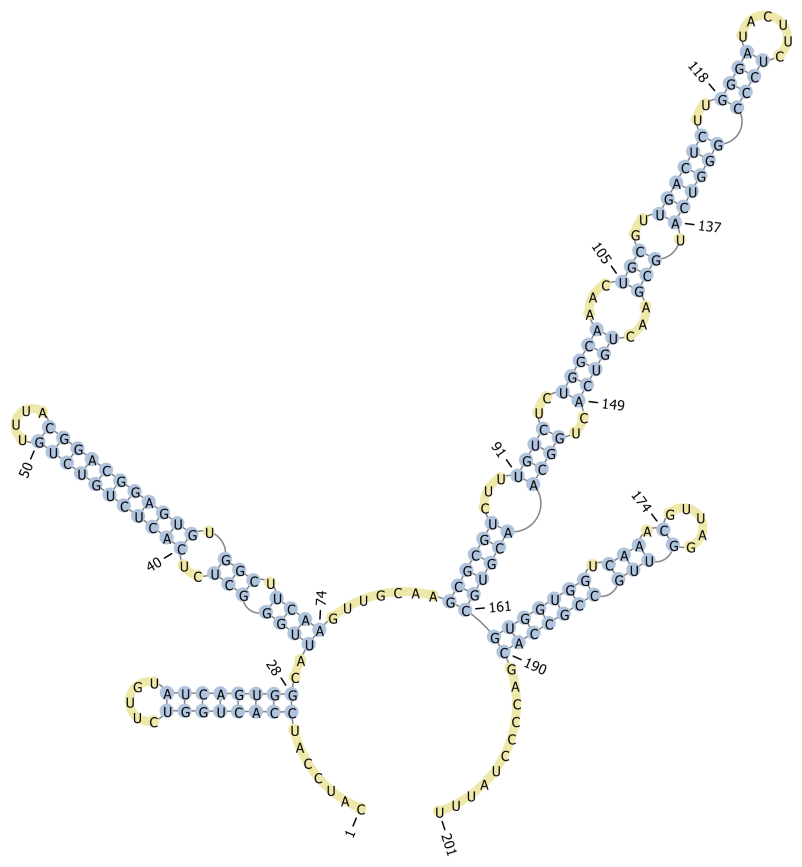


ITS2 secondary structure of *Cyclops kolensis*





ITS2 secondary structure of *Cyclops sternuus*


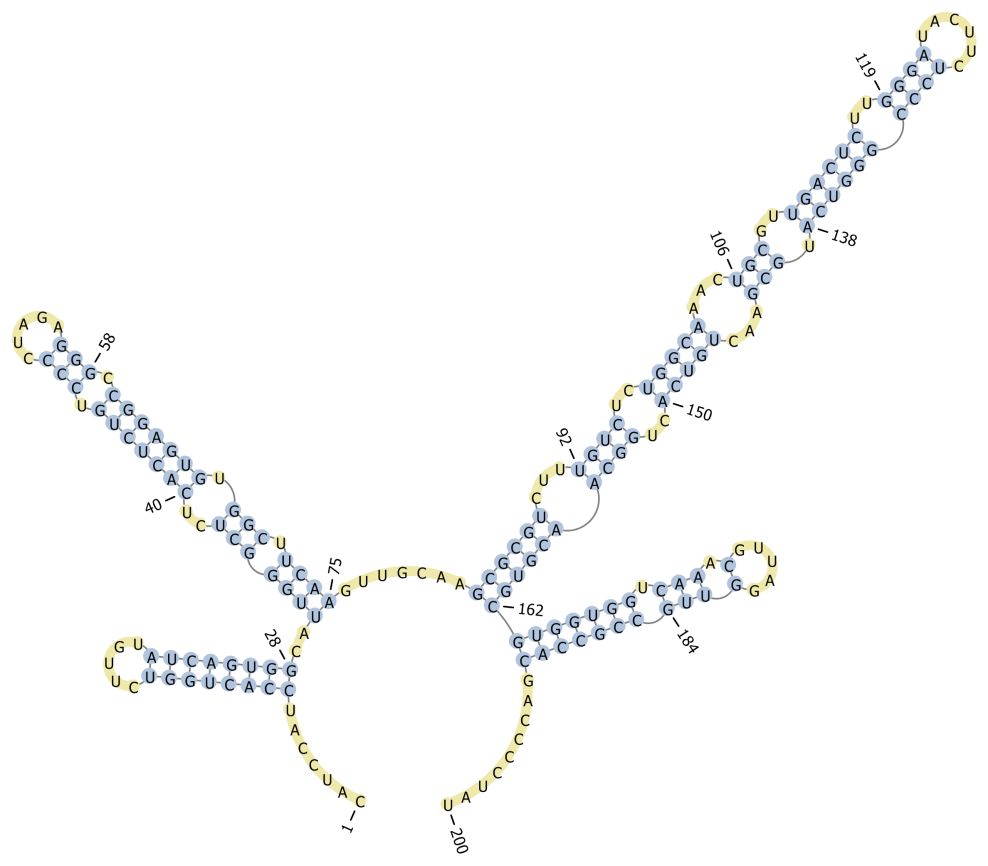


ITS2 secondary structure of *Diacyclops bicuspidatus*


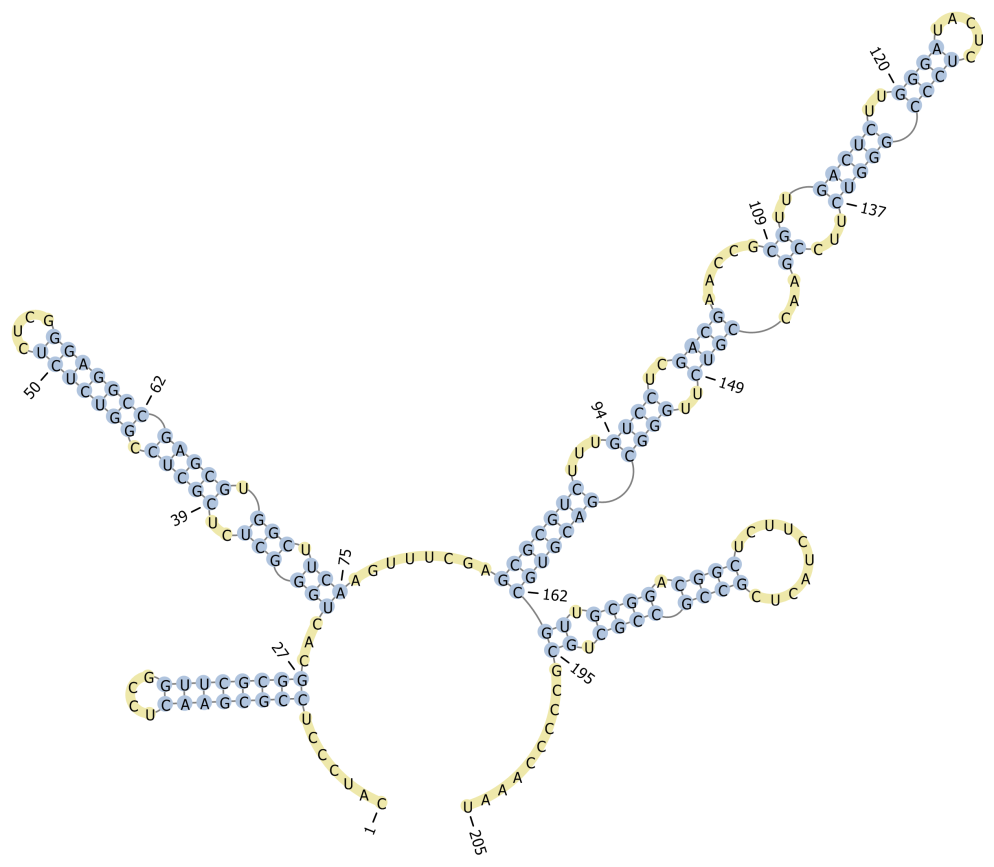


ITS2 secondary structure of *Macrocyclops albidus*


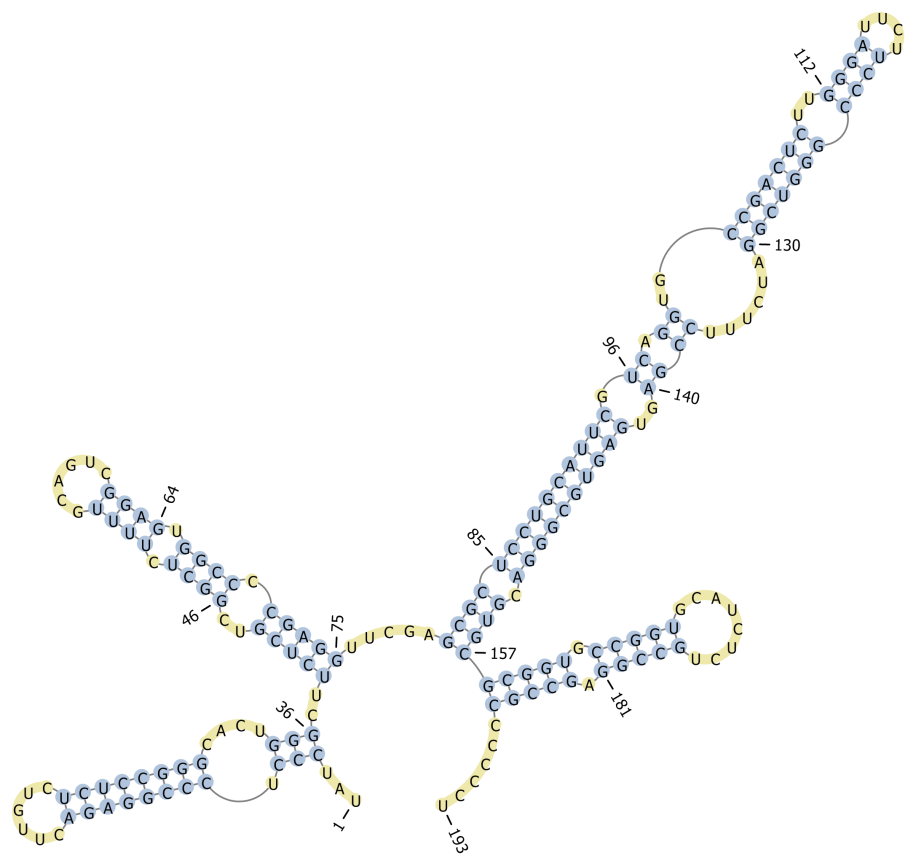


ITS2 secondary structure of *Macrocyclops distinctus*


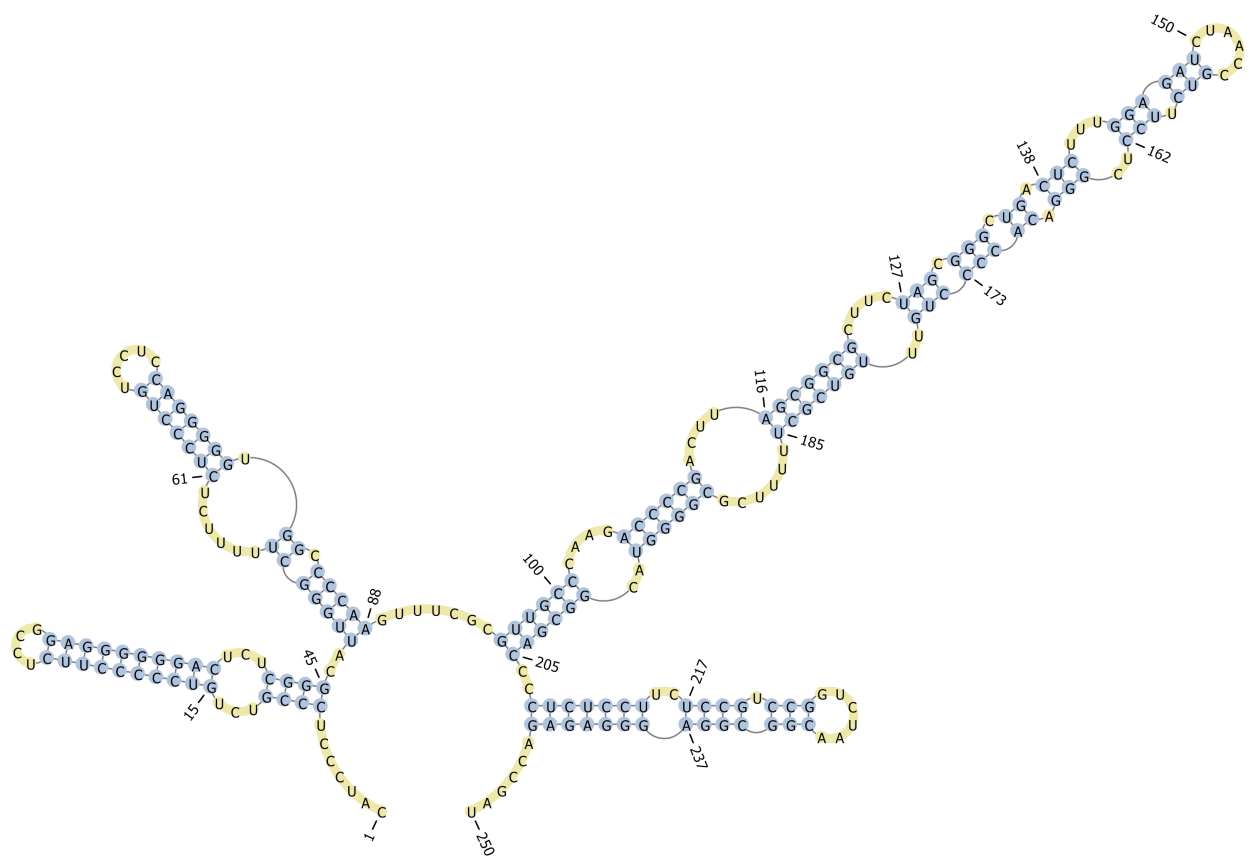


ITS2 secondary structure of *Megacyclops viridis* strain 1


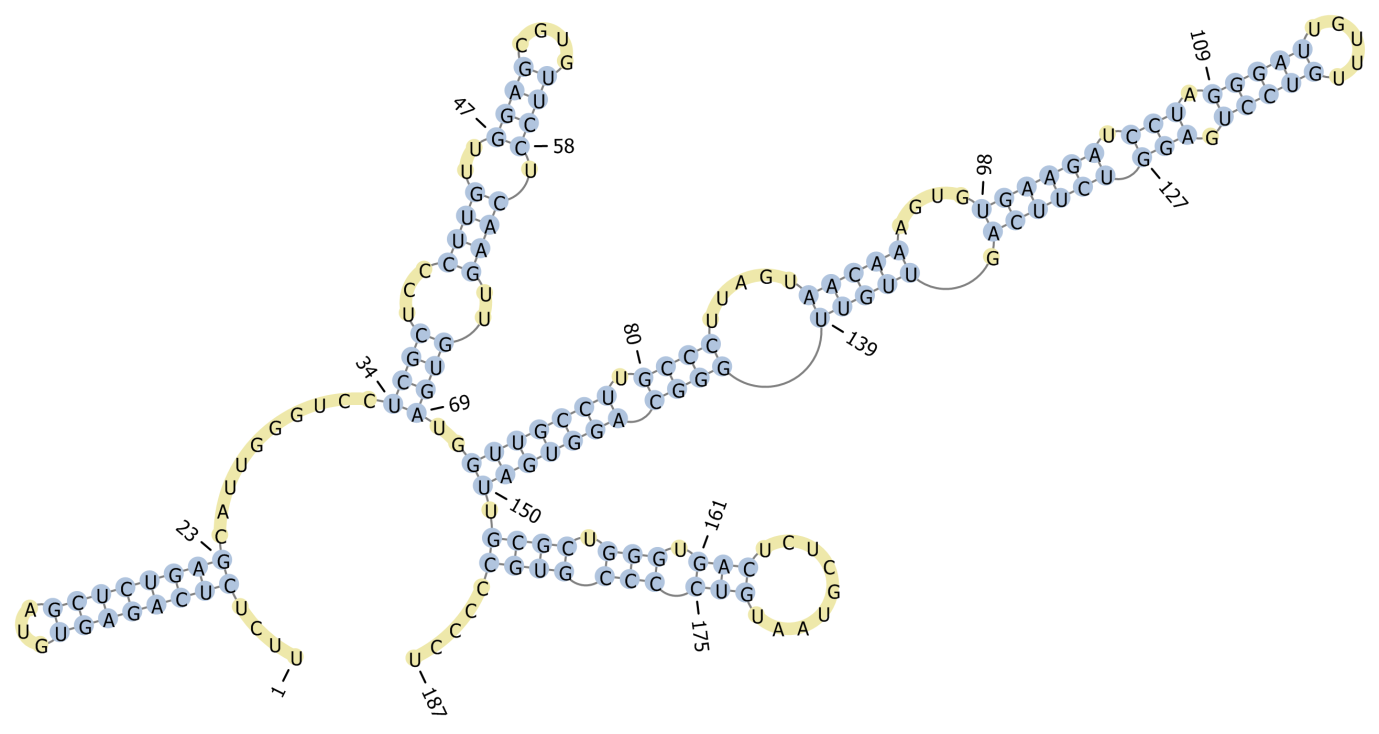


ITS2 secondary structure of *Megacyclops viridis* strain 2


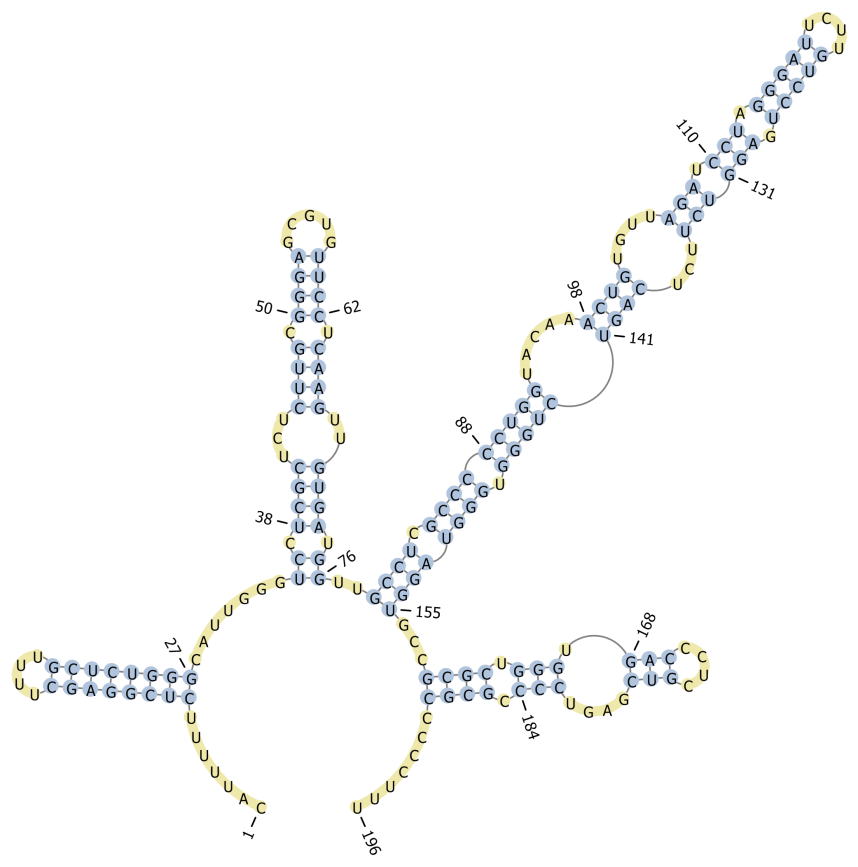


ITS2 secondary structure of *Mesocyclops leukarti*


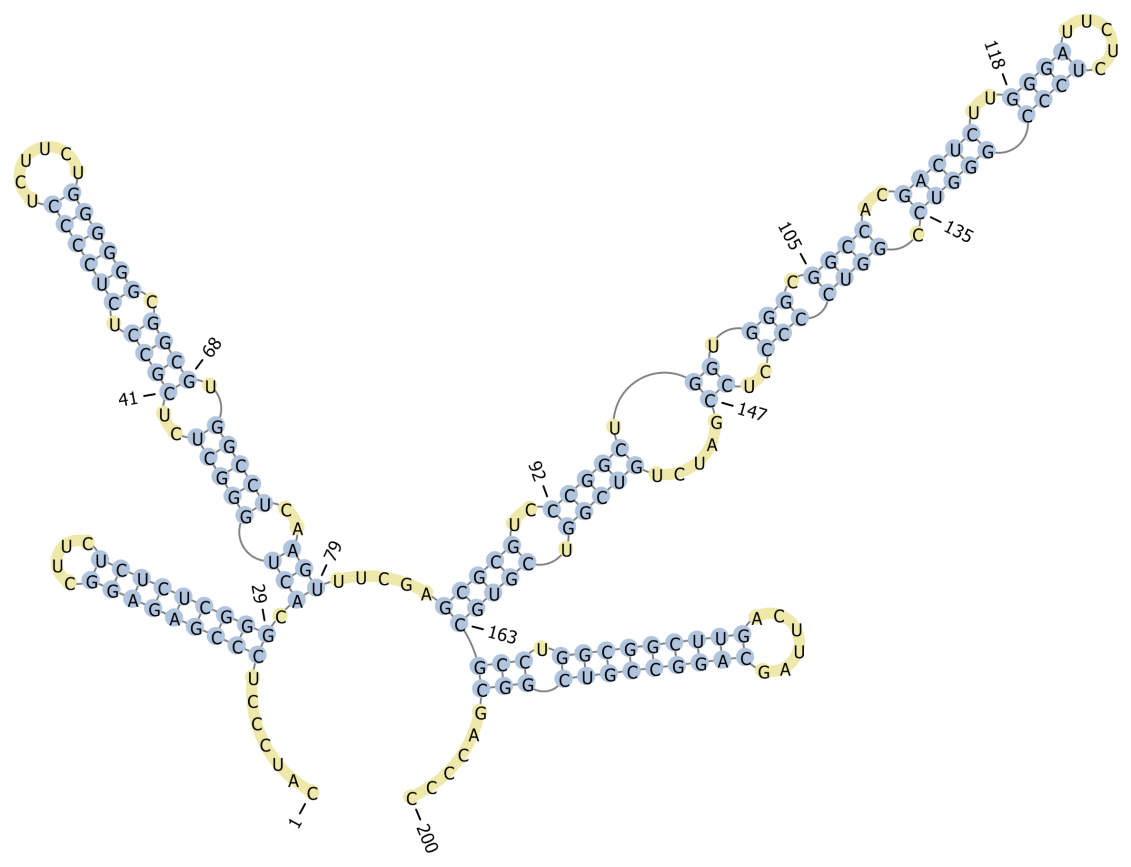


ITS2 secondary structure of *Oithona similis*


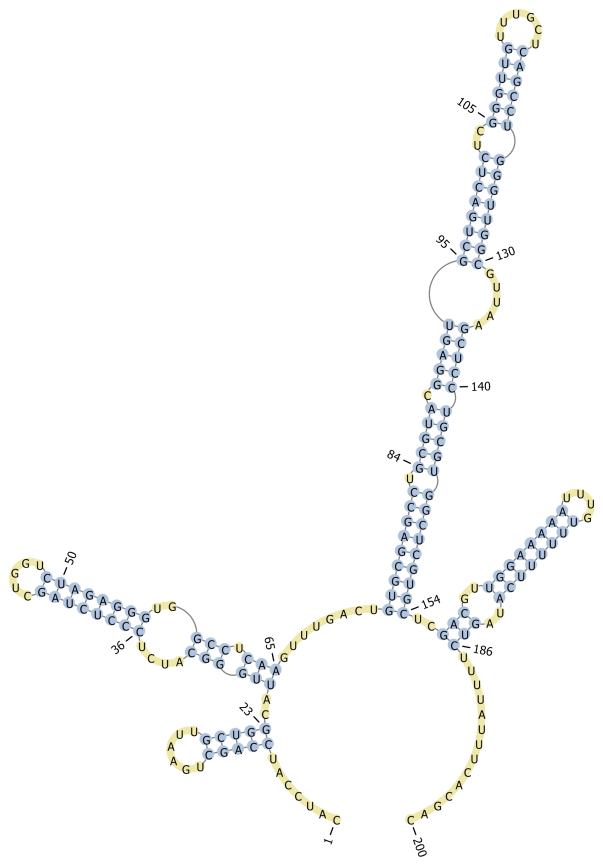


ITS2 secondary structure of *Oncaea* sp. strain MVZ-2013


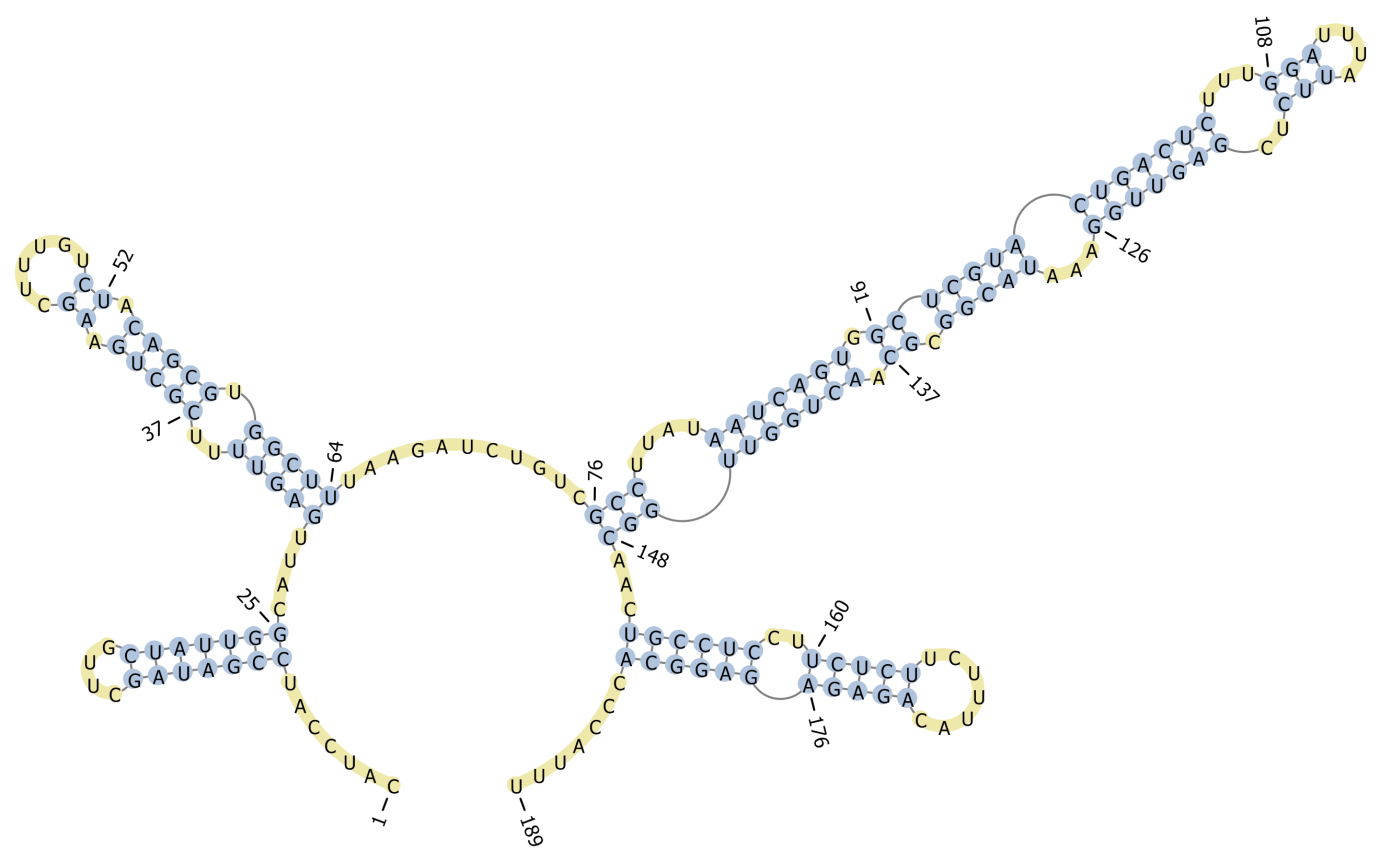


ITS2 secondary structure of *Thermocyclops crassus*


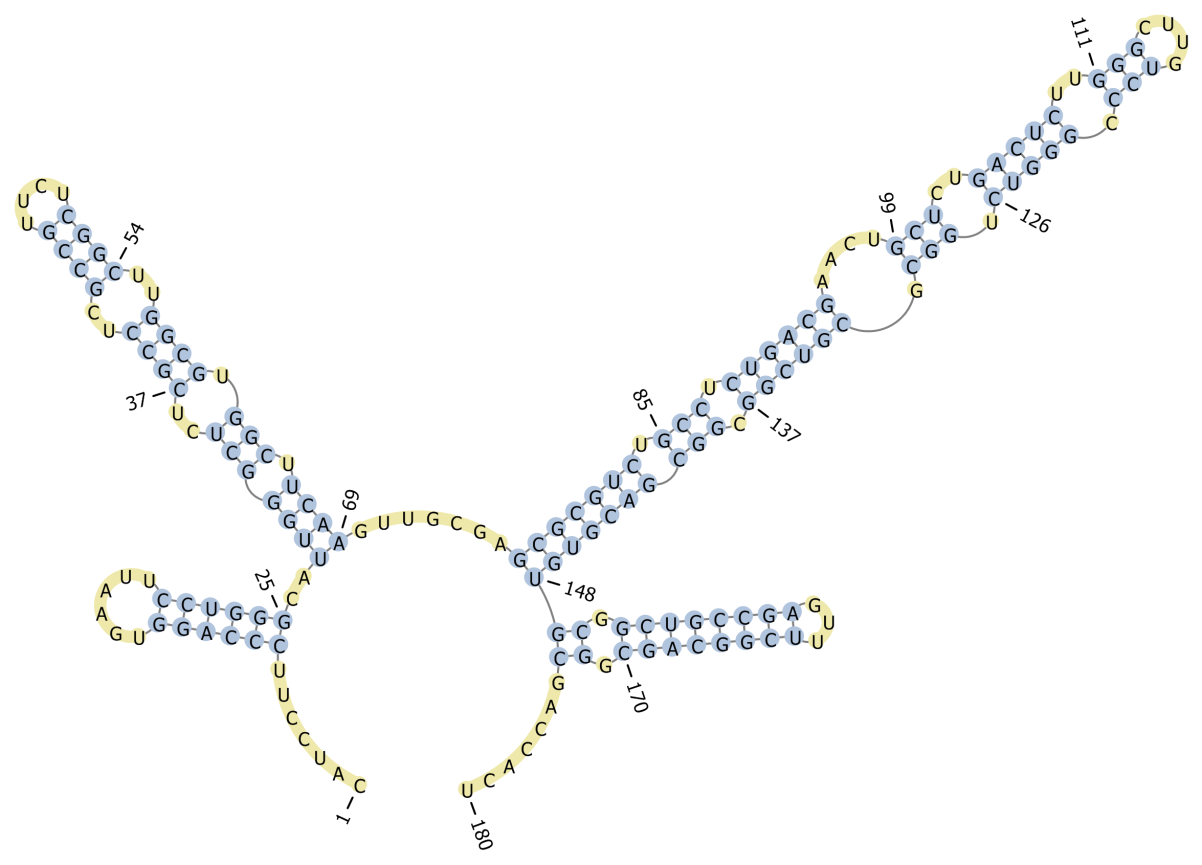


ITS2 secondary structure of *Thermocyclops oithonides*


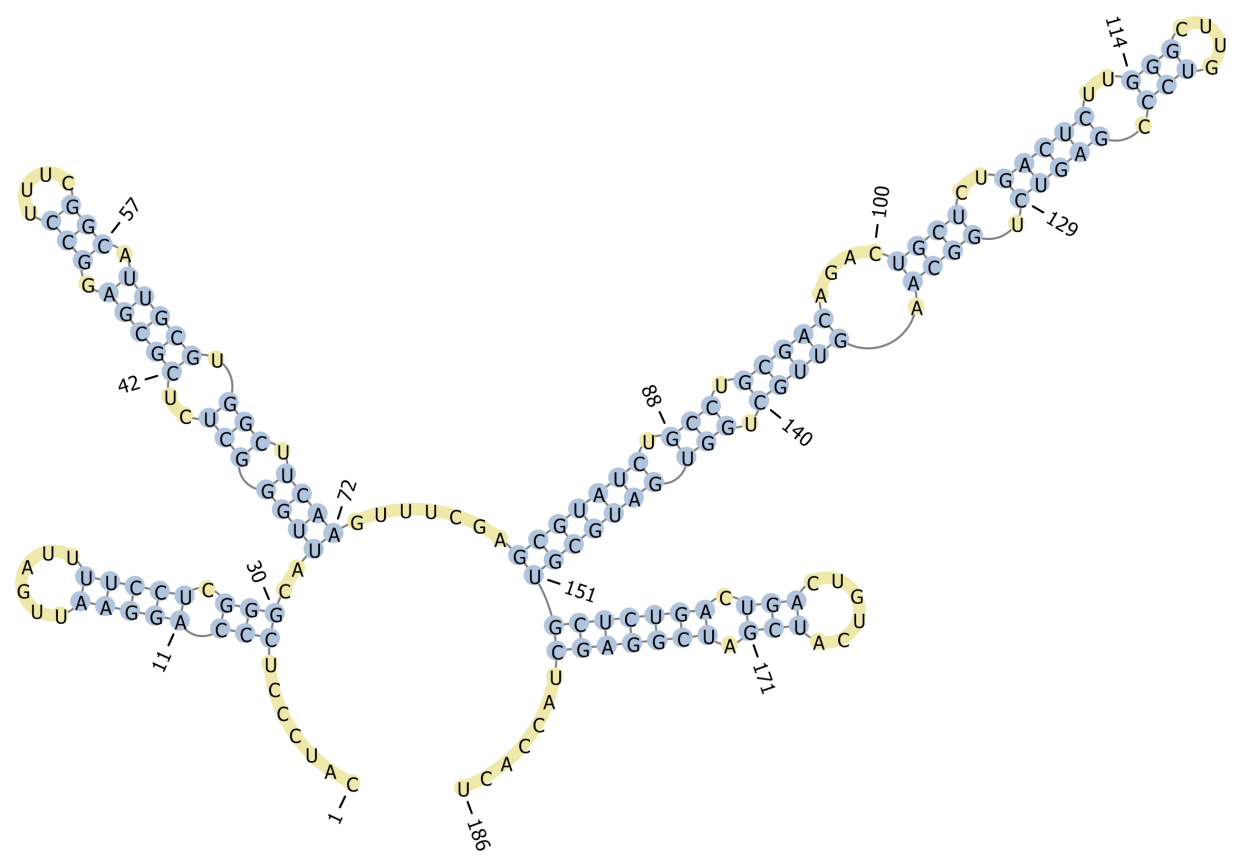

Supplement: S4 Fig — (DOCX) [file pone.0175662.s004.docx]
